# Supplementary material for: PREGO: A Literature and Data-Mining Resource to Associate Microorganisms, Biological Processes, and Environment Types
Source: Microorganisms. 2022 Jan 26;10(2):293. doi: 10.3390/microorganisms10020293 (PMC8879827; doi:10.3390/microorganisms10020293)
Supplement: Supplementary file 1 [file microorganisms-10-00293-s001.zip › microorganisms-1548015-supplementary.pdf]

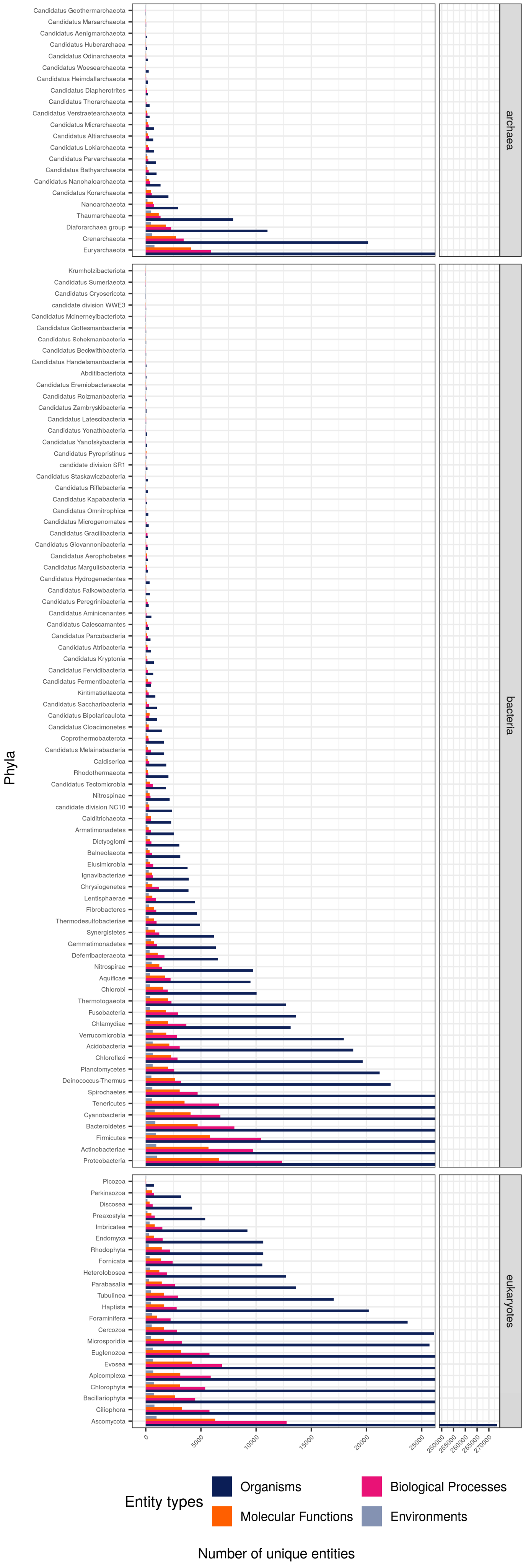

Figure S1: Summary of all the unique entities per phylum for each of the four entity types (in log10 scale) that appear in PREGO coming from the *Literature* channel.

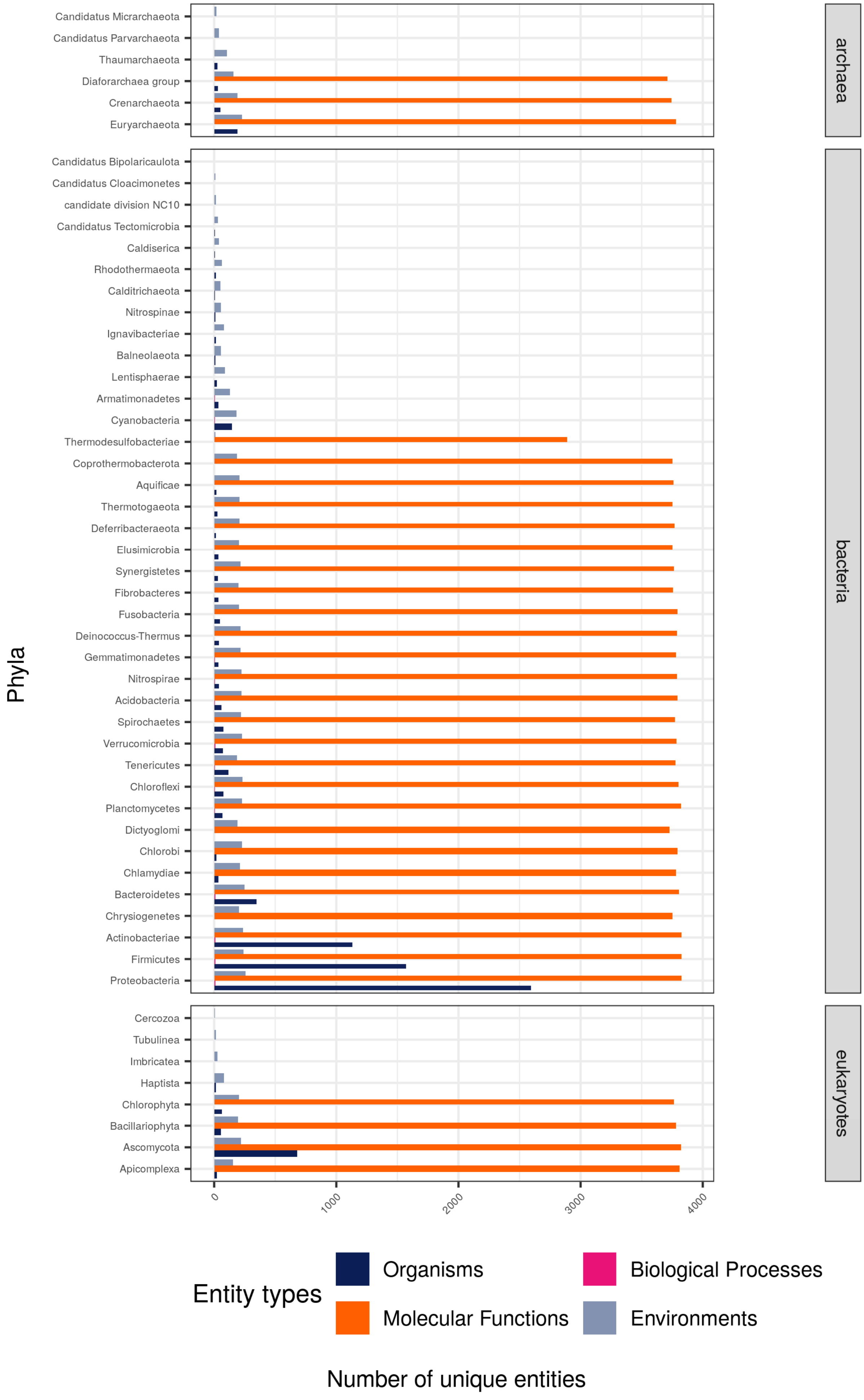

Figure S2: Summary of all the unique entities per phylum for each of the four entity types (in log10 scale) that appear in PREGO coming from the *Environmental samples* channel.

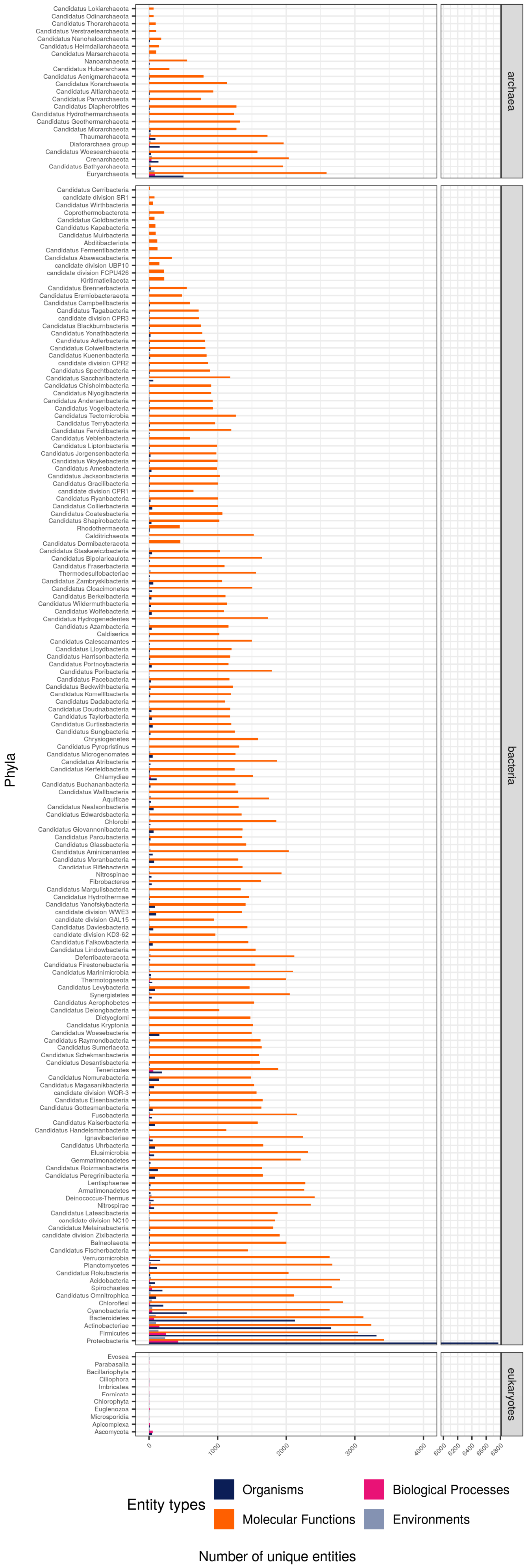

Figure S3: Summary of all the unique entities per phylum for each of the four entity types (in log10 scale) that appear in PREGO coming from the *Annotated genomes and Isolates* channel.
